# Economic Evaluations in Electrophysiology in the Last 15 Years: A Systematic Review of the Literature

**DOI:** 10.31083/RCM36206

**Published:** 2025-04-23

**Authors:** Davide Antonio Mei, Jacopo Francesco Imberti, Marco Vitolo, Niccolò Bonini, Edoardo Casali, Lucia Osoro, Ruben Casado-Arroyo, Giuseppe Boriani

**Affiliations:** ^1^Cardiology Division, Department of Biomedical, Metabolic and Neural Sciences, University of Modena and Reggio Emilia, Policlinico di Modena, 41121 Modena, Italy; ^2^Clinical and Experimental Medicine PhD Program, University of Modena and Reggio Emilia, 41121 Modena, Italy; ^3^Advocacy, Quality Improvement and Health Economics (AQIHEC) Committee, EHRA, 06903 Sophia Antipolis, France; ^4^Department of Cardiology, H.U.B.-Hôpital Erasme, Université Libre de Bruxelles, 1070 Bruxelles, Belgium

**Keywords:** atrial fibrillation, pacemaker, defibrillator, cost-effectiveness, health economics, ablation

## Abstract

**Background:**

Electrophysiology (EP) procedures, including cardiac implantable electronic devices (CIEDs) and ablations, are widely used to manage arrhythmias and heart failure. These interventions, though effective, require substantial resources, prompting the need for systematic economic evaluations to inform healthcare decision-making.

**Methods:**

A systematic review of studies from 2007 to 2024 was conducted in two phases. Phase one assessed trends in economic evaluations of EP procedures, analyzing 129 studies across regions and timeframes. Phase two focused on cost-effectiveness analyses of implantable cardioverter defibrillators (ICDs), cardiac resynchronization therapy defibrillators (CRT-Ds), and atrial fibrillation (AF) ablation, examining outcomes like quality-adjusted life years (QALYs) and incremental cost-effectiveness ratios (ICERs), while identifying factors influencing economic results.

**Results:**

EP procedures generally demonstrated favorable cost-effectiveness, particularly in high-income regions. Studies on ICDs and CRT-Ds consistently supported their economic value for patients with arrhythmias or heart failure, while AF ablation showed potential for long-term benefits, particularly when compared to medical therapies. However, results varied by region, reflecting differences in healthcare systems, costs, and patient populations.

**Conclusions:**

The review highlights the overall cost-effectiveness of EP procedures in many settings but underscores the need for tailored economic evaluations in low- and middle-income countries. Simplified methodologies and greater attention to regional contexts are recommended to guide resource allocation and policy development globally.

## 1. Introduction

Electrophysiology (EP) encompasses a range of specialized procedures that are widely used in modern cardiology practices, with applications that play a crucial role in the treatment and management of arrhythmias and heart failure [1, 2, 3, 4]. These procedures generally fall into two primary categories: (i) the implantation of cardiac implantable electronic devices (CIEDs), and (ii) the ablation of various arrhythmias (EP procedures). CIED procedures include devices like implantable cardioverter defibrillators (ICDs), pacemakers, and cardiac resynchronization therapy (CRT) devices, each targeting specific arrhythmia-related conditions or heart failure mechanisms. EP procedures focus on ablative techniques designed to interrupt abnormal electrical pathways, primarily to treat atrial fibrillation (AF) and other tachyarrhythmias [5]. The clinical application of these technologies has been thoroughly established through numerous clinical guidelines developed by leading cardiology societies worldwide [6]. These guidelines, grounded in high-quality evidence from clinical trials and observational studies, provide structured recommendations that aid in selecting the most appropriate procedure for each patient profile, significantly enhancing patient outcomes and reducing mortality in several clinical settings [6].

With the aging population and growing incidence of cardiovascular disease globally, the demand for both CIED and EP procedures is expected to rise, leading to an increased volume of these interventions performed in clinical practice [7]. However, the cost associated with each procedure is substantial and raises questions about healthcare sustainability, especially when implementing advanced and resource-intensive technologies. Despite strong evidence supporting improved patient outcomes, it is essential to understand whether these procedures are cost-effective in diverse healthcare systems. Assessing the economic impact of these interventions, specifically their cost-effectiveness or cost-utility, is critical for healthcare policymakers to allocate resources efficiently [8, 9].

Over the past 15 years, numerous studies have evaluated the economic aspects of EP and CIED procedures, offering insights into both their clinical benefits and cost implications. Given the extensive amount of recent research, our goal was to systematically review and summarize these cost-effectiveness analyses to provide an updated overview of the economic value of EP procedures in electrophysiology. In particular, our study aims to evaluate the distribution and volume of economic analyses for CIED and EP procedures across different geographic regions. In addition, as ICD, CRT, implants and AF ablation procedures are the most commonly performed procedures and represent significant expenses within electrophysiology, we specifically focused on these interventions to assess their cost-effectiveness in greater depth.

## 2. Methods

### 2.1 Search Strategy

A comprehensive and systematic literature search was conducted in PubMed. The initial search covered studies published from January 2007 to June 2023. Given the passage of time and the rapid development in the field of electrophysiology, an updated search was subsequently conducted to include studies published from July 2023 to October 2024.

The search strategy was developed to capture a broad spectrum of studies related to cost-effectiveness in electrophysiology and was designed using a combination of Medical Subject Headings (MeSH) terms and relevant keywords, such as “Implantable cardioverter defibrillator”, “cardiac resynchronization therapy”, “atrial fibrillation”, “ablation”, “tachycardia”, “pacemaker”, “cost-utility”, “cost-effectiveness”, and “cost-minimization”. Two independent reviewers (DAM. and JFI) screened all titles and abstracts identified from the searches to ensure accuracy and minimize selection bias. In cases where there was disagreement between reviewers, a consensus was reached through discussion or by consulting a third reviewer (GB).

### 2.2 Eligibility Criteria and Data Extraction

To structure our analysis effectively, we adopted a two-step approach for study selection based on inclusion criteria that varied by analysis phase.

**First Step:** In this initial step, we performed a broad systematic review to capture the full scope of economic evaluations in electrophysiology across continents and over time. The inclusion criteria were purposefully broad to ensure a comprehensive capture of available studies. This phase included:

Any form of economic analysis: studies encompassing cost-effectiveness, cost-utility, or cost-minimization analyses were included.

All EP and CIED procedures: including both ablation and implantation interventions.

During this phase, two reviewers (DAM. and JFI) extracted data on region, year of publication, and type of economic analysis conducted.

**Second Step:** In the second phase, a narrower focus was applied to provide in-depth insights into three high-impact interventions: ICD implantation, cardiac resynchronization therapy defibrillator (CRT-D) implantation, and AF ablation. The selection criteria for this phase were stricter to ensure consistency in evaluating cost-effectiveness measures.

Specific Economic Analysis Types: only cost-utility and cost-effectiveness analyses were included.

Outcome Measures: studies reporting quality-adjusted life years (QALYs), life-years (LYs), and incremental cost-effectiveness ratios (ICERs) were included.

Data extraction in this phase covered additional parameters, including time horizon, derivation cohort, analysis perspective, and patient population characteristics, alongside the specified outcomes.

The data from both phases were synthesized using a narrative approach. Structured tables were created to display the primary characteristics and outcomes of each study, allowing for a clear comparison of findings. Results were divided and reported separately for each major procedure category (ICD, CRT-D, and AF ablation).

## 3. Results

### 3.1 Overview of Included Studies

**Fig. 1.**
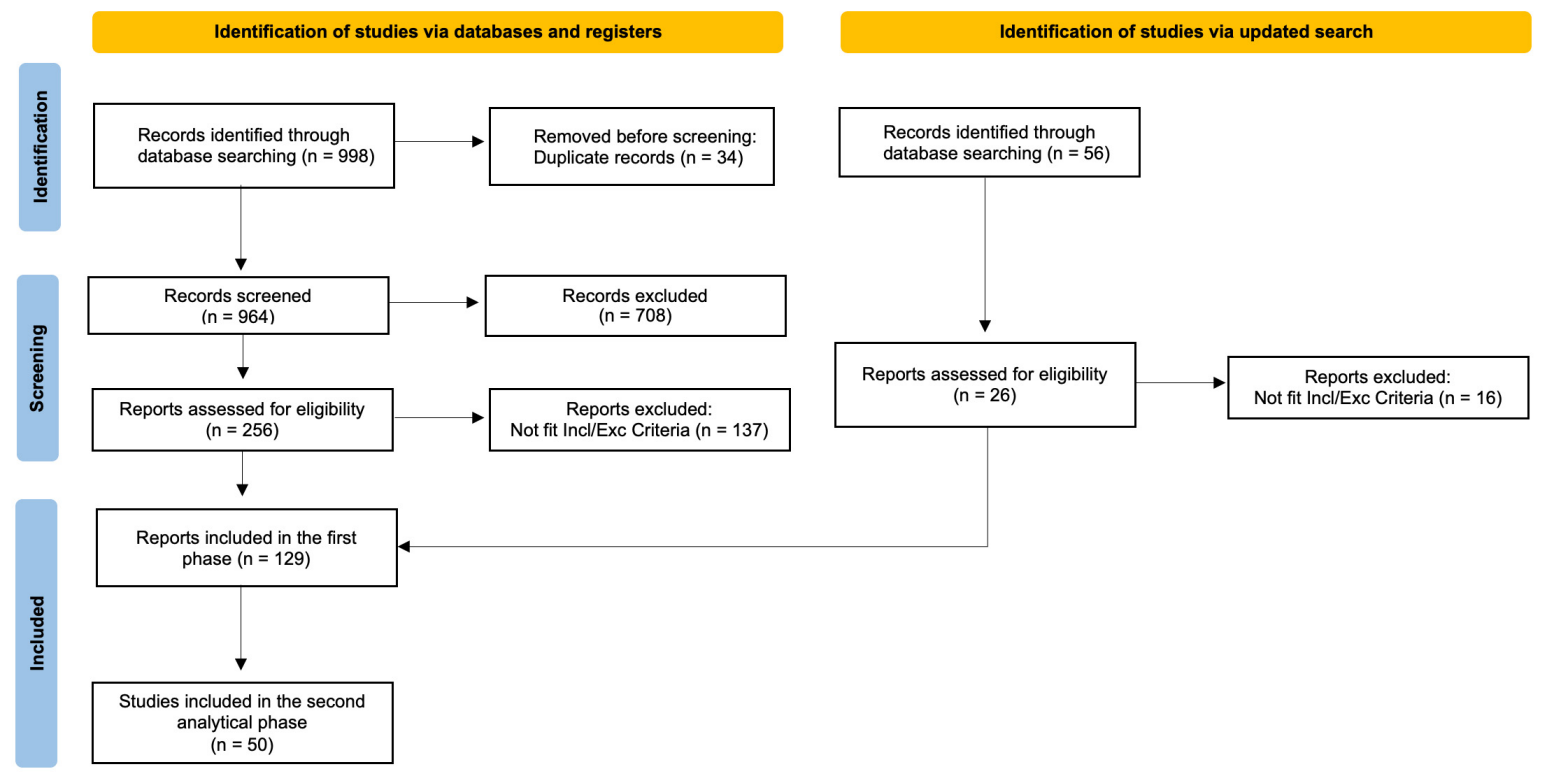
**PRISMA Flow-Chart of the Study**. Incl/Exc, inclusion/exclusion.

Our systematic literature search identified a total of 998 records. After screening titles and abstracts, 708 records were excluded due to irrelevance based on predefined inclusion criteria. A total of 256 full-text articles were reviewed for eligibility. We added 26 articles from the updated search, and as a result, we included a total of 129 articles for the first phase of the systematic review. From these, 50 studies were examined in the second phase using more stringent criteria (see Fig. 1).

### 3.2 Economic Analysis Across Procedures

**Fig. 2.**
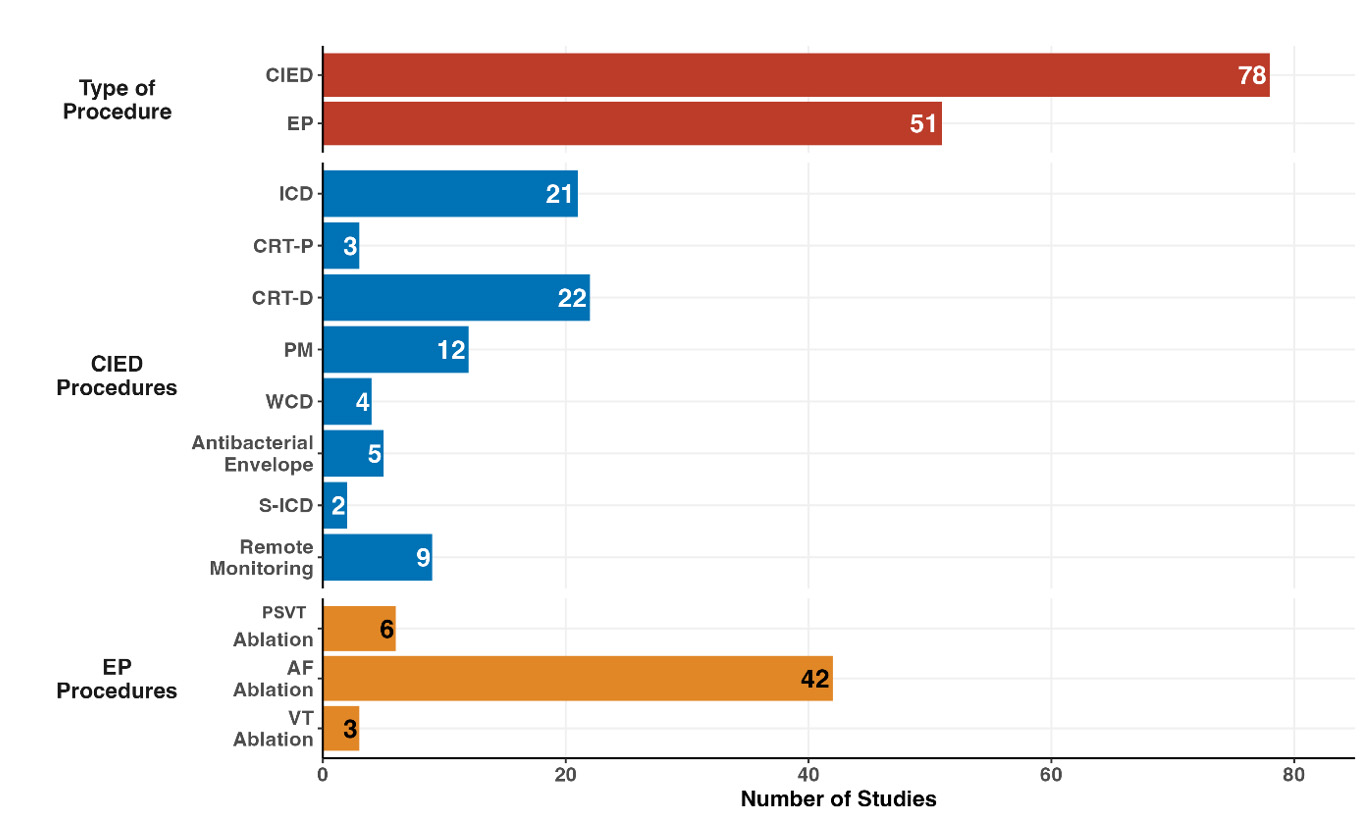
**Distributions of economic studies according to category of electrophysiological procedures**. EP, electrophysiology; CIED, cardiac implantable electronic device; ICD, implantable cardioverter defibrillator; CRT-D, cardiac resynchronization therapy with defibrillator; CRT-P, cardiac resynchronization therapy with pacemaker; AF, atrial fibrillation; PM, pacemaker; WCD, wearable cardiac defibrillator; PSVT, paroxysmal supraventricular tachycardia; VT, ventricular tachycardia; S-ICD, subcutaneous implantable cardiac defibrillator.

The results of the first phase are summarized in Fig. 2. When analyzing the types of procedures, 60.5% of the studies focused on CIEDs, with the remaining 39.5% addressing EP procedures. Within CIED studies, the majority evaluated CRT-D (22 studies; 28.2%) and ICD implantation (21 studies; 26.9%). For EP procedures, AF ablation accounted for the majority (42 studies; 82.3%), followed by other procedures, such as supraventricular tachycardia (6 studies; 11.8%) and ventricular tachycardia (3 studies; 5.9%). Remote monitoring of devices has been studied in 9 publications (6.7%), reflecting an emerging focus on leveraging technology to optimize outcomes.

**Fig. 3.**
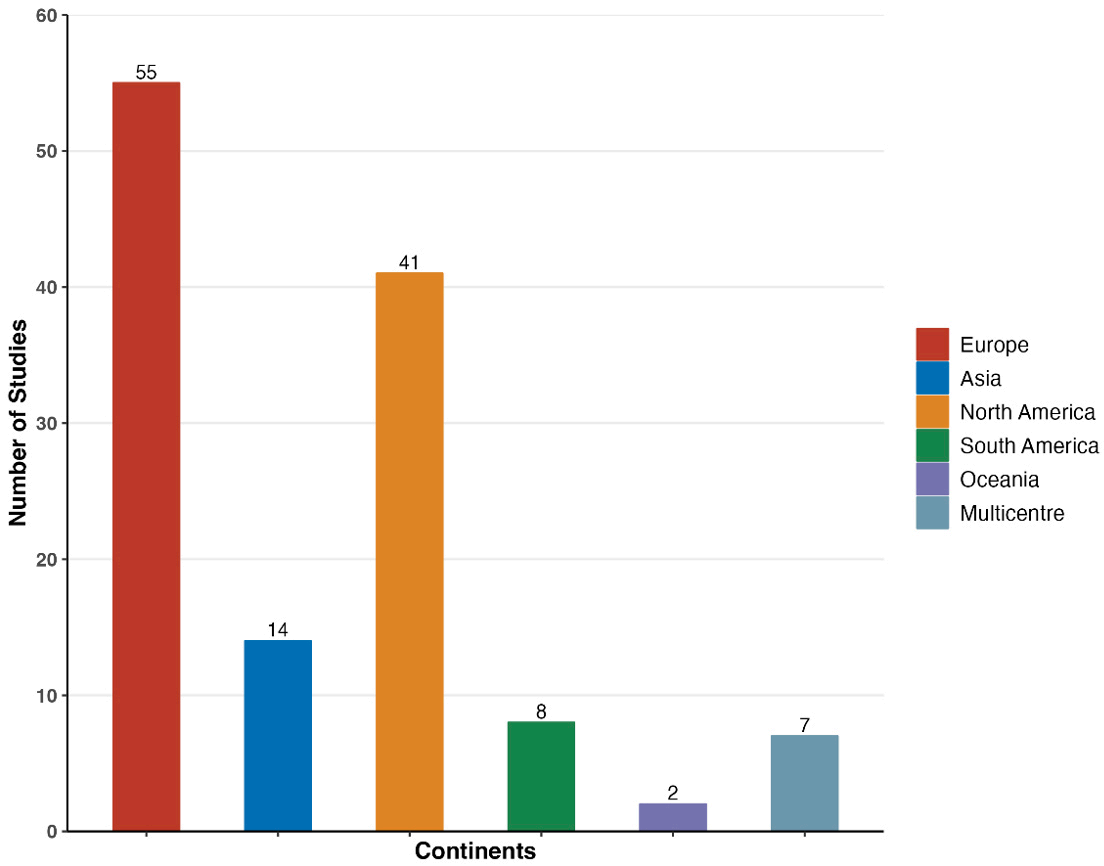
**Distributions of economic studies according to geographical setting**.

Economic evaluations of electrophysiology procedures continued to be predominantly conducted in high-income regions (Fig. 3). The geographical distribution of studies shows that Europe (55 studies; 43.3%) and North America (41 studies; 32.3%) remain the leading contributors, collectively accounting for 75.6% of all publications. This is followed by Asia (14 studies; 11.0%), South America (8 studies; 6.3%), Oceania (2 studies; 1.6%), and multicenter studies (7 studies; 5.5%).

### 3.3 Temporal Trends

**Fig. 4.**
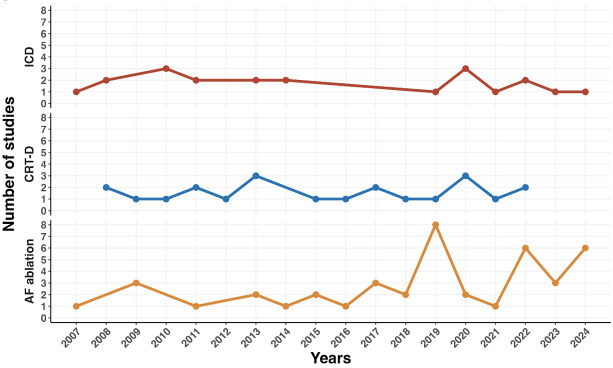
**Temporal trends of procedures related to electrophysiological procedures**.

A temporal analysis of publication frequency reveals several important trends (see Fig. 4). Studies evaluating ICD and CRT-D procedures have shown a relatively stable output over the past 15 years, reflecting sustained interest in these devices’ clinical and economic benefits. On the other hand, studies focusing on AF ablation have increased significantly since 2019, reaching a peak in 2024 with the publication of 6 studies in the final months of this review period. This trend aligns with the growing use of AF ablation in clinical practice and its recognition as a cost-effective treatment option for managing arrhythmias.

However, there was a sharp decline in economic evaluations in 2021, with publication numbers dipping across all categories. Despite this, a recovery in publication rates has been evident in subsequent years.

### 3.4 Focus on AF Ablation, ICD and CRT-D

#### 3.4.1 Cost-Utility and Cost-Effectiveness Analysis of Atrial Fibrillation Ablation 

A total of 21 studies were included in the analysis of AF ablation (Table 1, Ref. [10, 11, 12, 13, 14, 15, 16, 17, 18, 19, 20, 21, 22, 23, 24, 25, 26, 27, 28, 29, 30]), with 17 of these being cost-utility analyses [10, 11, 12, 13, 14, 15, 16, 17, 18, 19, 20, 21, 22, 23, 24, 25, 26] and 4 being cost-effectiveness studies [27, 28, 29, 30]. Of these, 18 studies demonstrated an economic advantage for AF ablation compared to conventional therapies, though the ICERs varied significantly across regions due to differences in healthcare costs, currency valuations, and local medical practices.

**Table 1.** **Summary of cost-effectiveness analyses for atrial fibrillation treatment modalities**.

Study	Year	Horizon	Region	Data derivation	Perspective	Population	Threshold	Results
Hansen *et al*. [10]	2024	40 years	Denmark	Cryo-FIRST; STOP AF First, EARLY-AF	Healthcare	PAF	€23,200	First-line Cryoablation vs AAD
								ICER $14,628/QALY.
								99.9% probability cost-effective
Andrade *et al*. [11]	2024	40 years	Canada	Cryo-FIRST; STOP AF First, EARLY-AF	Healthcare	PAF	$50,000	First-line Cryoablation vs AAD
								ICER $20,326/QALY.
								99.9% probability cost-effective
Paisey *et al*. [12]	2024	40 years	UK	Cryo-FIRST; STOP AF First, EARLY-AF	Healthcare	PAF	£20,000	First-line Cryoablation vs AAD
							£30,000	ICER £3783/QALY.
								89.5% probability cost-effective
								94.3% probability cost-effective
Wazni *et al*. [13]	2023	40 years	USA	Cryo-FIRST; STOP AF First, EARLY-AF	Healthcare	PAF	$50,000	First-line Cryoablation vs AAD
							$100,000	ICER $24,637/QALY.
							$150,000	76.2% probability cost-effective
								91.6% probability cost-effective
								95.2% probability cost-effective
Berman *et al*. [14]	2023	5 years	USA	ATTEST trial	Payer	PAF	$100,000	RF ablation vs AAD.
								ICER of $5298/QALY.
								98% probability cost-effective
Kim *et al*. [15]	2023	20 years	Korea	National Health Insurance (NHI) claims database of the Republic of Korea	Healthcare	CABANA like AF patients	$32,000	RF ablation vs AAD
								ICER $4739/QALY
Leung *et al*. [16]	2022	Lifetime	UK	Previous publication, with systematic review of the literature	Healthcare	AF patients	£20,000	AF ablation vs AAD
								ICER £6438/QALY
Chew *et al*. [27]	2022	5 years	USA	CABANA trial	Healthcare	AF patients	$100,000	AF ablation vs AAD
								ICER $57,893/QALY
								75% probability cost-effective
Hu *et al*. [28]	2022	Lifetime	China	ATTEST trial	Health care	AF patients	$20,390	RF ablation vs AAD
								ICER of $5972/QALY
								Cryoablation vs AAD
								ICER of $12,167/QALY
Lau *et al*. [17]	2021	30 years	Canada	CASTLE-AF	Healthcare	AF patients with HF	$CAD 50,000	AF ablation vs AAD
								ICER $CAD 35,360/QALY
								90% probability cost-effective
Chew *et al*. [29]	2020	Lifetime	USA	CASTLE-AF	Healthcare	AF in HF patients	$50,000	RF ablation vs AAD
								ICER $38,496/QALY
								75% probability cost-effective
Du *et al*. [18]	2019	8 years	China	Retrospective registry	Third-party payer	AF patients	NR	RF ablation vs AAD:
		15 years						8 years: ICER ¥66,764/QALY
		20 years						15 years: ICER ¥36,280/QALY
								20 years: ICER ¥29,359/QALY
Gao and Moodie [30]	2019	Lifetime	Australia	Review of the literature	Healthcare	AF in HF patients	$50,000	RF ablation vs AAD:
								ICER $55,942/QALY
Ming *et al*. [19]	2019	Lifetime	China	Retrospective registry	Healthcare	AF patients	NR	Cryoablation vs RF ablation: ICER $16,590/QALY
Sun *et al*. [20]	2019	10 Years	China	Retrospective registry	Payer	PAF	$25,305	Cryoablation vs RF ablation: ICER $35,060/QALY
Baykaner *et al*. [21]	2018	3 years	USA	CONFIRM	Payer	AF patients	$100,000	FIRM+PVI vs PVI alone:
								ICER $34,452/QALY
								75% probability cost-effective
Aronsson *et al*. [22]	2015	Lifetime	North Europe	MANTRA-PAF	NR	AF patients	€50,000	RF ablation vs AAD:
								ICER €50,570/QALY
Reynolds *et al*. [23]	2014	5-years	UK	STOP-AF	Payer	PAF	£20,000	Cryoablation vs AAD:
							£30,000	ICER £21,957/QALY
							£40,000	40% probability cost-effective
								86% probability cost-effective
								97% probability cost-effective
Blackhouse *et al*. [24]	2013	5-years	Canada	5 RCTs	Healthcare	AF patients	$50,000	AF ablation vs AAD:
							$100,000	ICER $59,194/QALY
								89% probability cost-effective
								90% probability cost-effective
Reynolds *et al*. [25]	2009	5-years	USA	Registries and RCT	Healthcare	PAF	NR	RF ablation vs AAD:
								ICER $51,431/QALY
McKenna *et al*. [26]	2009	Lifetime	UK	Metanalysis of different studies	Healthcare	AF patients	£20,000	RF ablation vs AAD:
								ICER £7910/QALY
								98% probability cost-effective

Legend: AAD, antiarrhythmic drugs; HF, heart failure; ICER, incremental cost-effectiveness ratio; NR, not reported; PAF, paroxysmal atrial fibrillation; PVI, pulmonary vein isolation; QALY, quality-adjusted life year; RCT, randomized controlled trial; RF, radiofrequency; $CAD, Canadian dollars; FIRM, focal impul and rotor modulation. €1 ≈ $1.0945. £1 ≈ $1.2961. ¥1 ≈ $0.1373.

Common comparators to AF ablation were antiarrhythmic drugs (AAD) and alternative ablation techniques, such as cryoablation versus radiofrequency (RF) ablation. RF ablation, in particular, was shown to have favorable ICERs in studies conducted in regions including China and the USA, with ICERs ranging from $5972/QALY [28] to $57893/QALY [27], indicating a high probability of cost-effectiveness for RF ablation relative to cryoablation within regional willingness-to-pay (WTP) thresholds, favoring its use as a first-line interventional treatment in appropriately selected patients with AF.

Further studies comparing AF ablation to AADs observed notable improvements in QALYs alongside reduced long-term healthcare costs, particularly in cases of paroxysmal AF, where ablation leads to substantial reductions in arrhythmia recurrence and associated healthcare use. Recent studies comparing cryoablation to AAD have provided additional insights into its economic value. For example, in Denmark [10], cryoablation was found to have an ICER of $14,628/QALY, with a 99.9% probability of being cost-effective compared to AAD as a first-line therapy. Similarly, a Canadian study [11] reported an ICER of $20,326/QALY, with a 99.9% probability of being cost-effective for cryoablation compared to AAD in patients with paroxysmal AF. In the UK [12] cryoablation showed an ICER of £3783($4720)/QALY, with a 89.5%–94.3% probability of being cost-effective depending on the WTP threshold.

Additional studies, such as one from the USA [13], demonstrated that cryoablation could be a cost-effective alternative to AAD with an ICER of $24,637/QALY, with a 76.2%–95.2% probability of being cost-effective at various WTP thresholds.

#### 3.4.2 Cost-Utility and Cost-Effectiveness Analysis of ICD 

A total of 14 studies were included in the analysis of ICDs (Table 2, Ref. [31, 32, 33, 34, 35, 36, 37, 38, 39, 40, 41, 42, 43, 44]), with 11 cost-utility analyses [31, 32, 33, 34, 35, 36, 37, 38, 39, 40, 41] and 3 cost-effectiveness studies [42, 43, 44]. The primary indication for ICDs was either primary or secondary prevention of sudden cardiac death in high-risk patients, including those with underlying cardiac conditions such as previous myocardial infarction or high New York Heart Association functional classification (NYHA class).

**Table 2.** **Summary of cost-effectiveness analyses for ICDs**.

Study	Year	Horizon	Region	Data derivation	Perspective	Population	Threshold	Results
Sun *et al*. [31]	2024	Lifetime	China	Chinese registry	Healthcare	High risk patients with DCM in primary prevention	CNY 85,698	ICD vs OMT
							CNY 257,094	ICER 139,652 CNY/QALY.
								92.1% probability cost-effective
Ribera *et al*. [32]	2022	Lifetime	Spain	CAT, DEFINITE; DANISH and SCD-HeFT	Healthcare	NI-DCM and I-DCM	€25,000	ICD vs OMT in I-DCM
								ICER €19,171/QALY
								ICD vs OMT NI-DCM
								ICER €31,084/QALY
Higuera *et al*. [33]	2021	Lifetime	Colombia	Improve SCA and from literature	Payer	High risk patients with DCM in primary prevention	Colombia: $58,903,902	ICD vs no ICD
			Mexico				Mexico: $594,383	Colombia: ICER $46,729,026/QALY
			Uruguay				Uruguay: $1,802,860	Mexico: ICER $246,016/QALY
								- Uruguay: ICER $1,214,937/QALY
Magnusson and Wimo [34]	2020	12 years	Sweden	Region Gävleborg	Healthcare	HCM	€53,050	ICD vs no ICD: ICER €15,150/QALY
Holbrook *et al*. [35]	2020	Lifetime	Taiwan	Improve SCA and from literature	Payer	High risk patients with DCM in primary prevention and in general DCM	NT $2,100,000	ICD vs no ICD
								High risk
								ICER: NT$441,153/QALY
								Primary prevention
								ICER NT$708,711/QALY
Atehortúa *et al*. [36]	2019	10 years	Colombia	Review of the literature	Healthcare	DCM	$19,139	ICD vs OMT
								ICER $13,187/QALY
Smith *et al*. [37]	2013	Lifetime	Netherlands	Metanalysis of 5 RCT	Payer	DCM NYHA class I, II and III	€80,000	ICD vs OMT
								ICER €43,9993/QALYs
								65% probability cost-effective
Gandjour *et al*. [38]	2011	8-years	Germany	MADIT-II	Healthcare	HF in I-DCM	NR	ICD vs OMT
								ICER €44,736/QALY
Alcaraz *et al*. [39]	2011	Lifetime	Argentina	Data derived from different RCT	Healthcare	Patients at high risk of sudden death	NR	ICD vs OMT
								In all perspective ICD was cost effective
Sanders *et al*. [40]	2010	Lifetime	NR	Data derived from different RCT	Healthcare	HF patients with indication for ICD	NR	ICD vs OMT
								In different RCT, ICD have different ICER
Cowie *et al*. [41]	2009	Lifetime	Europe	Data derived from different RCT	Healthcare	HF patients with indication for ICD in primary prevention	€40,000	ICD vs OMT:
								ICER €31,717/QALY
								85% probability cost effective
Thijssen *et al*. [42]	2014	Lifetime	Netherlands	Registry of single center in Leiden	NR	DCM	€40,000	ICD vs OMT
								ICER €29,369/LY
								€35,154/QALY
Ribeiro *et al*. [43]	2010	20-years	Brazil	Systematic review	Healthcare	HF patients with DMC in NYHA class II-III	NR	ICD vs OMT
								ICER PPP$50,345/QALY PPP$44,304/LY
Neyt *et al*. [44]	2008	Lifetime	Belgium	SCD-HeFT	Healthcare	HF patients NYHA class II-III	€50,000	ICD vs OMT
								ICER €59,989/QALY
								15.5% probability cost-effective

Legend: DCM, dilated cardiomyopathy; I-DCM, ischemic dilated cardiomyopathy; LY, life-year; NI-DCM, non-ischemic dilated cardiomyopathy; NYHA, New York Heart Association; NT$, New Taiwan dollar; OMT, optimal medical therapy; PPP$, purchasing power parity dollars; CAT, the Cardiomyopathy Trial; SCD-HeFT, Sudden Cardiac Death in Heart Failure Trial; SCA, sudden cardiac arrest. NT$1 ≈ $0.0326.

Several of these studies found ICDs to be cost-effective compared to conventional therapies, including optimal medical therapy (OMT), with ICERs ranging from $13,187/QALY in Colombia [36] to €31,717($35,320)/QALY in Europe [41]. These ICERs fell within the acceptable WTP thresholds for each respective region.

A notable finding was that in high-risk patients with dilated cardiomyopathy (DCM) in primary prevention, the ICERs varied significantly. In China, a 2024 study [31] found that ICDs had an ICER of CNY139,652/QALY, with a 92.1% probability of being cost-effective. In Spain, a study [32] reported ICERs of €19,171($21,340)/QALY for ICDs in ischemic dilated cardiomyopathy (I-DCM) and €31,084($34,610)/QALY for non-ischemic DCM. Other studies from Sweden [34] and Taiwan [35] also showed favorable ICERs for ICD therapy, ranging from €15,150($16,880)/QALY to $441,153/QALY, with probabilities of being cost-effective above 90% in most cases.

#### 3.4.3 Cost-Utility and Cost-Effectiveness Analysis of CRT-D

A total of 15 studies were included in the analysis of CRT-D, with 6 cost-utility analyses [45, 46, 47, 48, 49, 50] and 9 cost-effectiveness studies [51, 52, 53, 54, 55, 56, 57, 58, 59]. Among these, several studies demonstrated a favorable economic profile for CRT-D compared to alternative treatment options, including cardiac resynchronization therapy with pacemaker (CRT-P), standalone ICDs, and OMT.

The ICERs for CRT-D compared to CRT-P varied across countries and healthcare systems. For example, in Germany [51], an ICER of €24,659($27,450)/QALY was found, while in the United States, the ICER averaged $43,678/QALY [46]. These ICERs are consistent with the national WTP thresholds, resulting in high cost-effectiveness probabilities—often exceeding 70%—for CRT-D use in patients with advanced heart failure. Further analyses revealed that CRT-D was economically advantageous when compared to ICDs alone, with incremental gains in QALYs, making it a valuable treatment for patients who benefit from both resynchronization and defibrillation (Table 3, Ref. [45, 46, 47, 48, 49, 50, 51, 52, 53, 54, 55, 56, 57, 58, 59]).

**Table 3.** **Summary of cost-effectiveness analyses for cardiac resynchronization therapy**.

Study	Year	Horizon	Region	Data derivation	Perspective	Population	Threshold	Results
Claridge *et al*. [45]	2018	5 years	UK	Prospective registry of 2 centers (UK and France)	NR	CRT-D patients with need for replacement	£20,000	Replacement with CRT-D vs CRT-P
								ICER £305,712/QALYs
								2% probability cost-effective
Gold *et al*. [46]	2017	Lifetime	USA	REVERSE trial	Healthcare	HF patients in NYHA class I/II	$50,000	CRT-D vs CRT-P
								ICER $43,678/QALYs
Bertoldi *et al*. [47]	2013	10-years	Brazil	Registry of single center Brazilian patients	Payer	HF patients	Int$ 31,689	CRT-D vs CRT-P:
								ICER Int$84,345/QALY
								CRT-D vs ICD:
								ICER Int$36,940/QALY
								CRT-D vs OMT
								ICER Int$15,723/QALY
Linde *et al*. [48]	2011	10-years	Europe	REVERSE trial	Payer	HF patients with DCM in NYHA class I and 2	€33,000	CRT-D vs OMT
								ICER €14,278/QALY
								79% probability of cost-effective
Callejo *et al*. [49]	2010	Lifetime	Spain	Systematic review	Healthcare	HF patients	NR	CRT-D vs CRT-P:
								ICER €53,547/QALY
Blomström *et al*. [50]	2008	Lifetime	Denmark, Finland, Sweden	CARE-HF	Healthcare	HF patients in primary prevention, NYHA class II-III	€50,000	CRT-D vs OMT
								DenmarkI CER €6493/QALY
								Finland €3571/QALY
								Sweden €4759/QALY.
								>99% Probability of cost-effective
Hadwiger *et al*. [51]	2021	20 Years	Germany	COMPANION, CARE-HF	Payer	DCM	€18,000	CRT-D vs CRT-P
								ICER €24,659/QALY
Shah *et al*. [52]	2020	Lifetime	USA	Data derived from 13 RCT	Payer	DCM	$100,000	Compared to CRT-P/ICD and OMT, CRT-D is the most cost-effective treatment across different subgroup of patients
Permsuwan *et al*. [53]	2020	Lifetime	Thailand	CARE-HF, MIRACLE, MUSTIC, and COMPANION	Healthcare	DCM	$5156	CRT-D vs OMT
								ICER $3362/QALY
								and $2469/LY
Woo *et al*. [54]	2015	Lifetime	USA	MADIT-CRT and RAFT	Societal	HF patients NYHA class I-II	$50,000	CRT-D vs ICD
							$100,000	ICER $59,500/LY and $61,700/QALY
							$150,000	31%, 79% ,93% Probability of cost-effective according to the thresholds
Almenar *et al*. [55]	2013	10-years	Spain	REVERSE trial	Healthcare	HF patients NYHA class I-II	€30,000	CRT-D vs OMT
							€35,000	ICER 18,430/LY
							€40,000	€21,500/QALY
								65, 72, 80% probability of cost-effective according to the thresholds
Noyes *et al*. [56]	2013	4-years	USA	MADIT-CRT	Payer	HF patients with DCM NYHA class I-II	$50,000	CRT-D vs ICD
							$100,000	ICER 58,330/QALYs
								$80,910/LY
								40%, 80% probability of cost-effective according to the thresholds
Poggio *et al*. [57]	2012	Lifetime	Argentina	MADIT-CRT and REVERSE	Payer	HF patients with DCM and NYHA class I-II	NR	CRT-D vs OMT
								ICER ID$38,005/LY and ID$34,185/QALY.
Maniadakis *et al*. [58]	2011	Lifetime	Greece	CARE HF	Payer	HF patients with DCM in NYHA class II-III	€25,000	CRT-D vs OMT
								ICER €6045/QALY and €6222/LY
								100% probability of being cost-effective
Aidelsburger *et al*. [59]	2008	2-years	Germany	Companion	Healthcare	HF patients in NYHA class III-IV	NR	CRT-D vs OMT:
								ICER €88,143/QALY and €193,996/LY

Legend: ID$, international dollar; Int$, international dollar adjusted for purchasing power parity. Int$1 ≈ $1.00.

Recent studies have also highlighted the value of CRT-D in specific patient subgroups. In Spain, Almenar *et al*. [55] found an ICER of €21,500($23,950)/QALY when comparing CRT-D with OMT, with a 65–80% probability of cost-effectiveness depending on the WTP threshold. In Thailand, a study by Permsuwan *et al*. [53] (2020) showed an ICER of $3362/QALY for CRT-D compared to OMT, further confirming its economic benefit in specific populations.

## 4. Discussion

The findings from our systematic review provide a comprehensive overview of the economic analyses related to EP procedures and CIEDs, with particular attention to ICDs, CRT-D and AF ablation. This discussion contextualizes these findings within the broader healthcare landscape, highlights trends in cost-effectiveness across procedures and regions, and explores factors influencing economic analyses in this area of cardiology.

### 4.1 Distribution and Trends in Economic Analyses

Our review shows a clear concentration of cost-effectiveness studies in high-income countries, particularly in Europe and North America, which reflects both the healthcare infrastructure and financial resources available for high-cost interventions. This geographical focus raises concerns about the generalizability of findings to middle- and low-income countries, where cardiovascular disease incidence is rising, yet healthcare budgets are more constrained. Additional analyses are needed to assess which EP and CIED procedures offer the best allocation of available resources, considering local budgets and disease prevalence.

While the methodologies for assessing cost-effectiveness are similar to those used in high-income countries, the thresholds for cost effectiveness have to be adapted to the gross domestic product (GDP) (usually thresholds corresponding to 1–3 X GDP per capita are used) [60]. Additionally, in low-income countries, underdiagnosis, limited access to healthcare resources, and unequal distribution of services complicate the application of standard economic models. These issues, alongside the affordability of technology and availability of trained healthcare providers, must also be considered to ensure that interventions align with the unique needs and constraints of these countries.

Over the past 15 years, economic evaluations of CRT-D and ICD implantation have maintained a relatively steady publication rate. This suggests that these technologies are well-established in terms of economic justification and clinical benefit, thus reflecting the maturity of these interventions in cardiology. Conversely, studies on AF ablation have significantly increased, particularly post-2019. This increase correlates with greater acceptance of AF ablation as an effective treatment for rhythm management and symptom control in select patients, thereby emphasizing the need for up-to-date economic evidence as these newer treatments gain traction in clinical guidelines [61].

### 4.2 ICD and CRT-D Cost-Effectiveness: Consistent Benefits Across Populations

Our review confirms that both ICDs and CRT-D devices are generally cost-effective, especially for patients at high risk of sudden cardiac death or those with advanced heart failure, where improvements in QALYs are substantial. The ICERs for these devices fall within or just above common WTP thresholds in most countries, supporting their use in high-risk groups. These findings are consistent with existing clinical guidelines that recommend ICDs for primary and secondary prevention of cardiac arrest and CRT-D for heart failure patients with low ejection fractions [62].

Of notice, the evidence summarized in Table 3 clearly indicates that CRT-D is consistently cost-effective when compared to OMT alone or to ICDs in patients who are appropriately selected based on clinical criteria. This consistent finding suggests that CRT-D offers both clinical and economic benefits in these patient populations. However, determining the cost-effectiveness of CRT-D when compared directly to CRT-P is a more nuanced issue. In fact, the ICERs in studies comparing CRT-D to CRT-P often vary significantly, making it difficult to draw definitive conclusions. This observation is further supported by the results of the DANISH trial [63], which pointed out the substantial challenges in establishing clear cost-effectiveness between these two devices. These findings underscore the critical importance of accurate patient selection in ensuring that the clinical and economic benefits of CRT-D are fully realized. Effective patient selection can optimize outcomes, not only improving patient health but also ensuring that healthcare resources are used in the most efficient and effective manner possible.

Interestingly, the studies indicate variability in ICERs for CRT-D across different healthcare systems, suggesting that both clinical outcomes and the local costs of care influence cost-effectiveness significantly. These findings highlight the importance of considering both direct medical costs and healthcare system factors (e.g., reimbursement policies, device costs) when evaluating the economic benefits of CRT-D and ICDs. Advancements in device programming to optimize pacing algorithms have also shown potential for improving patient outcomes and reducing healthcare costs, further supporting the cost-effectiveness of these devices [64, 65].

Infections related to device implantation remain a significant concern, as they can lead to hospitalizations, re-interventions, and prolonged treatment [66]. These complications can notably affect the overall cost-effectiveness of ICD and CRT-D devices. Recent studies have highlighted the growing interest in strategies to prevent device-related infections, particularly through the use of antibacterial envelopes. These envelopes, which surround the device, have been shown to reduce infection rates and improve patient outcomes [67]. Several of the studies considered in our review underscore the cost-effectiveness of this approach [68], suggesting that infection prevention strategies, including the use of antibacterial envelopes, should be an integral part of evaluating the economic outcomes of ICD and CRT-D implantation.

### 4.3 AF Ablation: Economic Profile in Selected Populations

A growing body of literature supports the economic value of AF ablation, especially in younger patients or those with persistent symptomatic AF who are not adequately managed by AADs. The high upfront cost of AF ablation is offset by long-term reductions in healthcare use, which include fewer emergency visits, hospitalizations, and medication requirements. Our review reveals that cost-effectiveness for AF ablation is particularly favorable in regions with higher baseline rates of healthcare utilization due to AF-related hospitalizations, suggesting that healthcare systems with higher rates of acute care may experience more significant cost savings from AF ablation.

However, the variability in ICERs across different ablation technologies and regions underscores the complexity of justifying this procedure economically in all contexts. Notably, RF ablation has demonstrated better cost-effectiveness than cryoablation in several studies, indicating that choice of ablation modality may impact the economic justification for AF treatment. As shown in Table 1, ablation generally proves more cost-effective compared to AADs, as it leads to long-term reductions in healthcare utilization. However, when comparing the two ablation techniques (RF and cryo), the cost-effectiveness becomes less clear and varies significantly. The differences in ICERs largely depend on the WTP threshold, with studies showing that at lower WTP values, the advantage of RF over cryoablation is diminished [13, 23]. This highlights the complexity of economic evaluations when comparing procedures with similar outcomes and costs. As a result, the choice between RF and cryoablation may not be as straightforward in terms of cost-effectiveness, and a careful consideration of local economic contexts and healthcare systems is necessary. In the future, tools powered by artificial intelligence (AI) could assist in optimizing decision-making by integrating patient-specific data and healthcare system variables, potentially enhancing the precision and efficiency of economic evaluations [69, 70].

Emerging technologies, such as pulsed-field ablation (PFA), represent a particularly interesting area for future economic evaluations. Clinical studies to date suggest that PFA demonstrates similar 12-month outcomes to RF and cryoablation, though with slightly higher upfront costs [71]. Observational data indicate that PFA has the potential to reduce complications associated with AF ablation [72]. While complications such as vascular trauma, bleeding, and cardiac tamponade remain rare, they can influence both cost-effectiveness and hospital length of stay [73, 74, 75]. The use of advanced techniques, such as ultrasound-guided vascular access, has further minimized these risks, emphasizing the importance of procedural optimization in improving overall outcomes [76].

Furthermore, the clinical complexity of AF patients—such as the presence of comorbidities like heart failure, peripheral artery disease, chronic obstructive pulmonary disease obesity [77, 78, 79, 80]—can significantly impact their prognosis [81] and the overall cost-effectiveness of ablation. These patients often face a higher risk of procedural complications, longer recovery times, and a greater need for ongoing healthcare resources, which can erode the long-term savings typically associated with ablation. Therefore, future economic studies should also account for the influence of these factors on both the short-term and long-term costs associated with AF ablation, to better identify which patient populations are most likely to benefit economically from this intervention.

### 4.4 Impact of Healthcare Contexts and Study Designs on Cost-Effectiveness

Differences in study design, patient populations, and healthcare perspectives (payer vs. societal) significantly influenced the outcomes of cost-effectiveness analyses. Studies from countries with publicly funded healthcare systems tended to adopt a payer perspective, focusing on direct costs, whereas studies from countries with more privatized healthcare systems were more likely to adopt a societal perspective, incorporating indirect costs such as productivity losses.

Another factor contributing to variability in cost-effectiveness outcomes is the time horizon of analysis. For instance, studies with longer time horizons often show more favorable cost-effectiveness outcomes due to the cumulative benefits of reduced arrhythmia recurrence and improved patient outcomes over time. These factors should be considered when interpreting ICERs and QALYs, as variations in perspective and time horizon can alter the perceived economic value of EP and CIED procedures [82].

### 4.5 Potential Impact of COVID-19 on Economic Evaluations and Clinical Prioritization

A noticeable reduction in cost-effectiveness publications in 2021 suggests that the COVID-19 pandemic may have impacted both the conduct and publication of economic evaluations. The pandemic likely influenced healthcare resource allocation and procedural volumes, potentially affecting the demand for and perceived value of EP and CIED interventions [83]. Additionally, COVID-19 may have prompted a reassessment of healthcare priorities, with resources temporarily redirected to address pandemic-related challenges. Future studies should consider the potential long-term impact of the pandemic on EP procedure accessibility, utilization, and economic outcomes.

In this context, remote monitoring of patients with CIEDs has gained increased attention, as it allows for continuous surveillance, reducing the need for in-person visits and potentially lowering costs associated with hospital readmissions or unplanned procedures [84]. Studies suggest that remote monitoring can improve clinical outcomes by enabling early detection of complications, such as device malfunctions or arrhythmias. However, its effectiveness depends on patient digital literacy, as those with lower levels of digital proficiency may face challenges engaging with the system [85]. Addressing these barriers is essential for maximizing the benefits of remote monitoring in diverse patient populations [86].

### 4.6 Study Limitations

This study has several limitations that should be acknowledged. We included only studies published in English, which may have excluded relevant studies in other languages, particularly those conducted in non-English-speaking countries. The concentration of studies in high-income countries limits the generalizability of findings to low- and middle-income countries, where healthcare infrastructure, costs, and patient populations may differ.

Another limitation is that economic studies may not always represent real-world scenarios. Also generalization in different settings is an issue due to differences in health care systems, protocols, patient populations, resources. In some studies, the data may be incomplete or difficult to capture like productivity losses due to the disease and intervention or other indirect costs.

Pricing is dynamic and devices or material for electrophysiological studies change over time due to market competition. On top of that, as it has been described above, variation in insurance coverage or government reimbursement policies can impact economic assessments.

Some of the publications described use decision-analytic models like Markov models, which require assumptions that can introduce bias. On top, there is a lack of a uniform methodology in conducting and reporting economic studies that could induce inconsistencies in results.

The heterogeneity in different clinical substrates, study designs, cost perspectives, and patient populations among the included studies may have introduced variability that could influence the results and make direct comparisons challenging. Some potentially valuable studies may have been excluded due to the strict focus on ICER, QALY, and LY outcomes, which may not capture the full economic or patient-centered value of EP and CIED interventions. Given the timeframe of our study, some recent innovations or procedural advancements in EP and CIED technology may not be represented, and the effects of COVID-19 may have skewed recent economic data due to temporary shifts in healthcare priorities.

Finally, rapid innovation in the field and fast learning curves of new procedures may change cost and improve outcomes in a short period of time.

Addressing all these limitations would require multidisciplinary approaches, integrating clinical expertise, robust economic modeling and real-world evidence collection to provide more comprehensive and meaningful insights with the objective to improve economic assessments.

## 5. Conclusions

Our systematic review underscores the interest in assessing the economic value and sustainability of several key electrophysiology procedures, notably ICD and CRT-D implantation and AF ablation, across a range of healthcare contexts. These interventions not only improve patient outcomes but also constitute solutions with a favourable cost-effectiveness profile for healthcare systems, particularly when deployed in high-risk populations. The review highlights that while upfront costs are substantial, the long-term economic benefits and improved quality of life make these interventions economically advantageous in many high-income countries.

However, the findings emphasize a need for region-specific economic analyses, especially in middle- and low-income countries where cardiovascular disease is rising, and healthcare resources are limited. Further research into cost-effectiveness in these regions, as well as studies considering alternative healthcare perspectives and indirect cost impacts, will be critical in guiding healthcare policy and resource allocation. As EP and CIED procedures evolve, ongoing economic evaluations will play an essential role in ensuring that these high-impact interventions are accessible, affordable, and sustainable for the populations who stand to benefit the most.

## Availability of Data and Materials

The data that support the findings of this study are available from the corresponding author, upon reasonable request, and after approval of all other co-investigators.

## Supplementary Material

Supplementary material associated with this article can be found, in the online version, at https://doi.org/10.31083/RCM36206.
